# Prostate cancer treatment does not increase the risk of cardiovascular events

**Published:** 2007

**Authors:** Makarand V. Khochikar

**Affiliations:** Department of Uro-oncology, Siddhi Vinayak Ganapati Cancer Hospital, Miraj, India. E-mail: khochikar@rocketmail.com

## SUMMARY

This paper is aimed at studying the risk of cardiovascular disease mortality in patients with prostate cancer and to compare this with the risk in the general population. The study population consisted of participants of the Rotterdam section of the ESRPC trial (European Randomized Study of Screening for Prostate cancer). Standardized mortality ratios (SMRs) of cardiovascular mortality for 2221 prostate cancer patients has been calculated in this study including the analysis for the treatment subgroups–radical radiotherapy (RT), radical surgery, watchful waiting and hormonal therapy. Cardiovascular mortality was defined as any deaths from cardiovascular disease, coronary artery disease, acute myocardial infarction, other diseases of the heart and cerebrovascular accidents. The treatment received by the prostate cancer patients was in the form of radical RT in 40.2%, radical prostatectomy in 30.3%, watchful waiting in 14.9% and hormonal therapy in 5.4% (unknown in 9.2%).

At mean follow-up of 5.5 years 12% of prostate cancer patients (258) had died. The SMRs for prostate cancer patients were found to be low in the range of 0.37-0.49 as compared to 0.90 in the general population. This low SMR was found in all patients irrespective of the treatment they had received. Therefore the authors have concluded that less emphasis should be put on risk of cardiovascular events as a contraindication for aggressive, especially surgical treatment for prostate cancer patients.

## COMMENTS

While counseling the patients for choosing the treatment options in prostate cancer we tend to give significant importance to life expectancy, existing co-morbid conditions such as cardiac diseases and also take into account the possibility of adverse cardiac events in the intra- postop period and the long term as well. Cardiovascular morbidity and mortality is always talked about in hormonal therapy. On this background, this study is an eye-opener. The authors have authentically proved that there is no increased cardiovascular mortality in these patients irrespective of the line of treatment. Satariano *et al* first coined this kind of thought in 1998.[1] There were no studies after this and I think the authors have successfully put this issue in the right perspective. This information though cannot be taken as the ‘final word’ on the issue of cardiac mortality in prostate cancer patients, it is certainly useful in understanding the fact that as believed in the past there is no increased mortality due to cardiac events. It's good information to use at the time of counseling the patients and certainly useful for the patients in decision-making.
